# Yield Losses Caused by Barley Yellow Dwarf Virus-PAV Infection in Wheat and Barley: A Three-Year Field Study in South-Eastern Australia

**DOI:** 10.3390/microorganisms9030645

**Published:** 2021-03-19

**Authors:** Narelle Nancarrow, Mohammad Aftab, Grant Hollaway, Brendan Rodoni, Piotr Trębicki

**Affiliations:** 1 Agriculture Victoria, Grains Innovation Park, Horsham, VIC 3400, Australia; mohammad.aftab@agriculture.vic.gov.au (M.A.); grant.hollaway@agriculture.vic.gov.au (G.H.); piotr.trebicki@agriculture.vic.gov.au (P.T.); 2 Agriculture Victoria, AgriBio Centre, Bundoora, VIC 3083, Australia; brendan.rodoni@agriculture.vic.gov.au; 3 School of Applied Systems Biology, La Trobe University, Bundoora, VIC 3083, Australia; 4 Faculty of Veterinary and Agricultural Sciences, University of Melbourne, Horsham, VIC 3400, Australia

**Keywords:** barley yellow dwarf virus, BYDV, wheat, barley, yield loss, vectors, aphids

## Abstract

Barley yellow dwarf virus (BYDV) is transmitted by aphids and significantly reduces the yield and quality of cereals worldwide. Four experiments investigating the effects of barley yellow dwarf virus-PAV (BYDV-PAV) infection on either wheat or barley were conducted over three years (2015, 2017, and 2018) under typical field conditions in South-Eastern Australia. Plants inoculated with BYDV-PAV using viruliferous aphids (*Rhopalosiphum padi*) were harvested at maturity then grain yield and yield components were measured. Compared to the non-inoculated control, virus infection severely reduced grain yield by up to 84% (1358 kg/ha) in wheat and 64% (1456 kg/ha) in barley. The yield component most affected by virus infection was grain number, which accounted for a large proportion of the yield loss. There were no significant differences between early (seedling stage) and later (early-tillering stage) infection for any of the parameters measured (plant height, biomass, yield, grain number, 1000-grain weight or grain size) for either wheat or barley. Additionally, this study provides an estimated yield loss value, or impact factor, of 0.91% (72 kg/ha) for each one percent increase in natural BYDV-PAV background infection. Yield losses varied considerably between experiments, demonstrating the important role of cultivar and environmental factors in BYDV epidemiology and highlighting the importance of conducting these experiments under varying conditions for specific cultivar–vector–virus combinations.

## 1. Introduction

Cereals, a staple food in many parts of the world, are continually threatened by abiotic (e.g., temperature, water, and nutrition stress) and biotic (e.g., weeds, pests, and diseases) factors. It has been estimated that plant diseases cost the global economy approximately USD 220 billion each year [1]. Diseases caused by viruses result in significant economic losses worldwide through crop failure and yield and quality losses, as well as increased input costs associated with disease management and prevention [2]. Barley, cereal, and maize yellow dwarf viruses (referred to collectively throughout this manuscript as YDVs) belong to the family *Luteoviridae* and are among the most widespread and important viruses affecting cereals worldwide. They commonly infect wheat (*Triticum aestivum*), barley (*Hordeum vulgare*), oat (*Avena sativa*), and other species belonging to the family *Poaceae*. Currently, ten barley yellow dwarf (BYDV), cereal yellow dwarf (CYDV), and maize yellow dwarf virus (MYDV) species are listed on the ICTV master species list: BYDV-kerII, BYDV-kerIII, BYDV-MAV, BYDV-PAS, and BYDV-PAV have been assigned to the *Luteovirus* genus; CYDV-RPS, CYDV-RPV, and MYDV-RMV have been assigned to the *Polerovirus* genus; and BYDV-GPV and BYDV-SGV have not been assigned to a genus [3]. BYDV-PAV, BYDV-MAV, CYDV-RPV, and MYDV-RMV have been found in Australia [4,5], where BYDV-PAV is considered the most abundant YDV species, particularly in the South-Eastern Australian state of Victoria [4,6,7,8,9]. YDVs are phloem-limited viruses which are persistently transmitted from infected to healthy plants by aphids [10,11]. The bird cherry-oat aphid (*Rhopalosiphum padi*) and the corn aphid (*Rhopalosiphum maidis*) are the most common vectors of YDVs in Australia [12].

Symptoms of YDV infection include yellowing and/or reddening of leaves, stunted growth, and reduced root biomass [11], however infection can also be symptomless. Numerous studies have reported significant yield losses due to YDV infection [13,14,15,16,17,18,19], and losses of up to 93% have been reported in field experiments after artificial inoculation [18,20]. In Australia, cereals such as wheat, barley, and oats are widely grown, and yield losses of up to 72 kg/ha have been reported for each 1% increase in virus incidence (percentage of plants infected), resulting in losses of up to 3790 kg/ha [17,19,21]. Symptoms and yield losses associated with YDV infection are usually more severe when plants are infected at the early growth stages [11,22,23,24]. The impacts of YDV infection can vary depending on factors such as virus species, host cultivar, plant growth stage at the time of infection, aphid vector, and environmental conditions [13,15,24,25,26].

Despite the implementation of the latest disease management strategies and an increased use of insecticides to control virus vectors over the past thirty years, YDVs still occur with high incidence in cereal fields in South-Eastern Australia, particularly in higher-rainfall regions [7,9]. Furthermore, a recent study found that YDVs were more prevalent, and occurred with higher incidence, in cereals in the region throughout 2014–2017 [7] than had previously been reported more than thirty years earlier [8]. Therefore, it is likely that yield losses associated with YDVs in South-Eastern Australia have been underestimated in recent years due to a lack of current yield loss data. 

Four field experiments were conducted over three years with varying seasonal conditions to quantify the effects of BYDV-PAV, the most prevalent YDV species found in cereals in South-Eastern Australia, on yield and yield components of wheat and barley that are currently grown in the region. This study aimed to provide current yield loss data that can be used to obtain a more accurate and updated estimation of the impact of YDVs in South-Eastern Australia. 

## 2. Materials and Methods

### 2.1. Field Sites and Experiments

**Figure 1:**
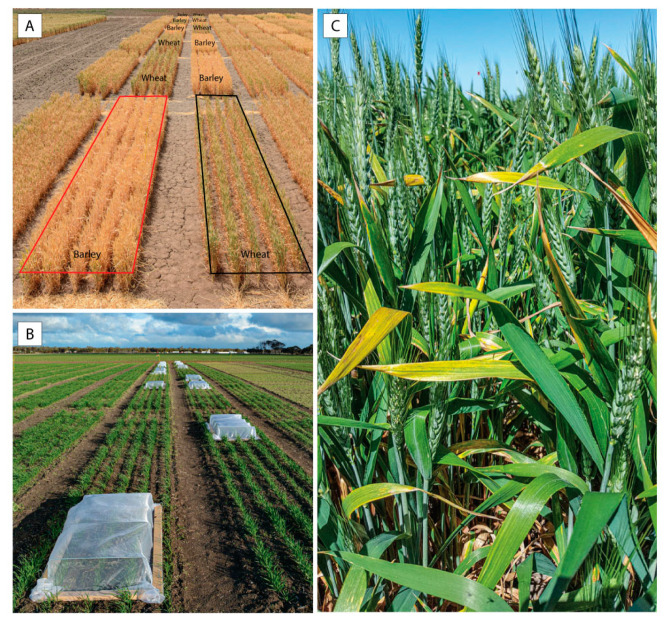
(**A**) The experimental layout in wheat and barley; (**B**) Field inoculation cages used to cover the virus-treated plots during inoculation; (**C**) Widespread leaf-yellowing symptoms of barley yellow dwarf virus (BYDV) background infection observed in non-inoculated control plots of wheat in 2017 in South-Eastern Australia.

Four field experiments to quantify the effects of BYDV-PAV infection on plant growth, yield, and yield components of field-cultivated wheat and barley under typical conditions were conducted at two sites in the Wimmera region, Victoria in South-Eastern Australia. Experiments 1 (2015), 2 (2015), and 3 (2017) were conducted at the Agriculture Victoria Plant Breeding Centre at Vectis (36°44′ S, 142°6′ E). Experiment 4 (2018) was conducted at the Agriculture Victoria Wimmera Research Station at Longerenong (36°40′ S, 142°18′ E). The Vectis and Longerenong field sites are both located in the Wimmera region and are approximately 18 km apart. The long-term (1990–2018) mean annual maximum temperature and mean annual rainfall of the Wimmera region were 21.6 °C and 404 mm, respectively (www.bom.gov.au; www.longpaddock.qld.gov.au/silo, accessed on 5 February 2021) [27]. Each experiment was designed using a randomized block design with 6 replicates (Figure 1A,B) and was direct-seeded using a cone seeder (PJ green, Grovedale, Australia) with 183 mm row spacing and a target establishment density of 150 plants/m^2^. The wheat cultivar Yitpi was evaluated in experiment 1, the barley cultivar Hindmarsh was evaluated in experiment 2, while the wheat cultivar Mace was evaluated in experiments 3 and 4. Yitpi, a commonly grown cultivar in the Wimmera region, has recently been replaced by Mace. Field sites were maintained using agronomic practices typical for the region.

### 2.2. Virus Propagation and Aphid Colony

The isolate of BYDV-PAV used to inoculate the virus-treated plots in each field experiment was collected from an infected oat plant in Horsham, Victoria, Australia with the virus identity confirmed by tissue blot immunoassay (TBIA) [9] and reverse transcription-polymerase chain reaction (RT-PCR) [28]. Viruliferous *R. padi* were maintained on infected wheat plants (cv. Yitpi) contained in cages in plant growth chambers at 20 °C with a 14:10 h light:dark photoperiod. Aphids were allowed a virus acquisition period of at least 7 days before they were used to inoculate plants in the field experiments.

### 2.3. Inoculation of Virus-Infected Plots with BYDV-PAV

In experiments 1 and 2, the three treatments were: (1) early BYDV inoculation (BYDV 1, inoculated at the seedling stage, Zadoks growth stage Z12 where two leaves had emerged) [29]; (2) later BYDV inoculation (BYDV 2, inoculated at the early tillering stage, Z22, where two tillers were visible); and (3) a non-inoculated control. In experiments 3 and 4, the two treatments were early BYDV inoculation (inoculated at the seedling stage, Z12) and a non-inoculated control. In each experiment, treatments were applied to plots 180 cm × 3 rows (1.62 m^2^) in size. Plots of wheat and/or barley selected for BYDV-PAV infection were inoculated with the virus by placing plant sections containing viruliferous *R. padi* alongside each row of plants within the plot. All plants within the plot were then covered with a large field cage (Figure 1B) for 3–5 days to contain the aphids and prevent virus contamination of control plots. Plants within each inoculated and control plot were sprayed with pyrethrum (Yates, active ingredient: pyrethrins) and Confidor (Bayer, active ingredient: imidacloprid) insecticides immediately after the cages were removed. All plots were then sprayed with insecticide as part of the normal spray program throughout the growing season.

### 2.4. Assessment of BYDV-PAV Incidence

In each experiment, 45–60 whole tillers were randomly collected from individual plants from each plot before maturity and tested for BYDV-PAV to assess inoculation success and levels of background infection using TBIA [9]. The number of positive tillers in the samples collected from each plot was recorded and the percentage of positive tillers was calculated.

### 2.5. Harvest Assessments

At plant maturity, the height of 12–15 plants in each plot was measured, recorded, and averaged. The above-ground portion of all plants from each plot was then hand harvested, placed into large paper bags, and transported to the laboratory, where plant biomass was measured. Samples were threshed using a Hans-Ulrich Hege 16 laboratory thresher (Wintersteiger, Ried im Innkreis, Austria). Grain was then aspirated with a vacuum separator (Kimseed, Wangara, Australia), counted using a Numigral seed counter (CHOPIN Technologies, Cedex, France), weighed, and 1000-grain weight was calculated. Grain size was assessed by passing each grain sample through 2.8 mm, 2.5 mm, and 2.2 mm sieves using a Sortimat laboratory sorting machine (Pfeuffer GmbH, Kitzingen, Germany) and calculating the percentage of grain in each range. Harvest index was calculated by dividing the grain yield (g) by plant biomass (g). In experiments 3 and 4, grain protein (%) and moisture content (%) were measured using a CropScan 3000B whole-grain analyzer (Next Instruments, Condell Park, Australia). Additionally, the number of heads in each plot was counted in experiments 3 and 4, then the total grain weight per head and the number of grains per head were calculated.

### 2.6. Weather Data

All rainfall and temperature data used to represent the Wimmera region, and therefore the Vectis and Longerenong field sites, were obtained from the Bureau of Meteorology (BOM) website (www.bom.gov.au) and SILO (www.longpaddock.qld.gov.au/silo) [27] from weather station number 79,028 (accessed on 5 February 2021), which was located in the paddock adjacent to the Longerenong field site. Long-term rainfall and temperature values were calculated using all available data from 1961–2018 while average annual temperature and rainfall data were used to demonstrate the variation in weather conditions in the Wimmera region in each year of the field study (Table 1).

### 2.7. Data Analysis

Grain weights from each plot were converted to grain yield (kilograms per hectare, kg/ha) prior to analysis. Yield losses (kg/ha) were calculated by subtracting the mean yield of the inoculated plots from the mean yield of the non-inoculated control plots with percentage yield loss also calculated. Means and standard errors of the means (SEMs) were calculated using GenStat 14th Edition (VSN International, Hemel Hempstead, UK). The normality of residuals was checked using quantile–quantile plots (Figures S1–S4). Analysis of variance (ANOVA) and Tukey’s honestly significant difference (HSD) test were used to analyze data from experiments 1 and 2. Experiments 3 and 4 consisted of two treatments, therefore paired, two-sided *t*-tests were used to analyze data from experiments 3 and 4 instead of ANOVA. The relationship between the natural BYDV-PAV background infection present in the non-inoculated control plots (x) and grain yield (y) in experiment 3 was tested for normality using the Shapiro–Wilk test and analyzed using polynomial linear regression and Pearson’s product moment correlation. Normality, ANOVA, *t*-tests, linear regression analysis, and correlations were performed using R version 4.0.0 (R Foundation for Statistical Computing, Vienna, Austria), and differences were considered statistically significant at *p* < 0.05. Figures were produced in SigmaPlot (Systat Software, San Jose, CA, USA).

## 3. Results

During the three years of this study, four individual field experiments were conducted to quantify yield losses associated with BYDV-PAV infection under varying seasonal conditions in the Wimmera region, South-Eastern Australia. Weather conditions varied between the 3 years of the study (Table 1), resulting in different rainfall, grain yield, green bridge, and background virus infection (Figure 1C) each year, and higher levels of BYDV-PAV background infection were observed in non-inoculated control plots of wheat in 2017 (22–60%) compared to 2015 (4–19%) and 2018 (3–17%).

### 3.1. Experiment 1 (2015); Wheat (cv. Yitpi)

**Figure 2:**
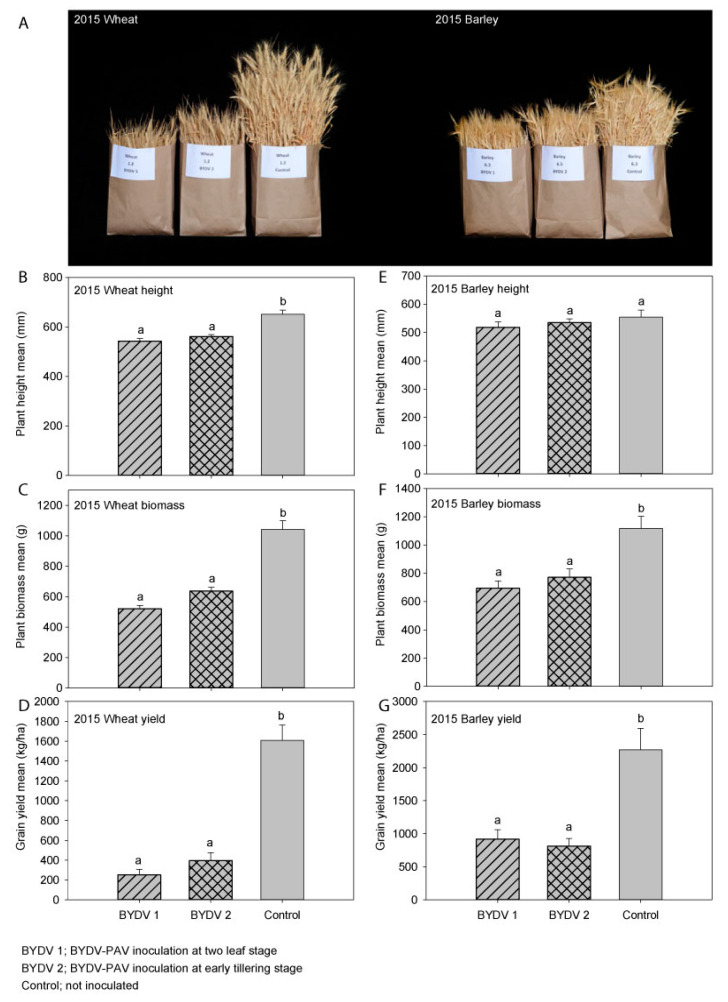
The effect of early (BYDV 1), later (BYDV 2), or no (Control) barley yellow dwarf virus-PAV (BYDV-PAV) inoculation on: (**A**) plant growth of wheat (left) and barley (right); (**B**) plant height; (**C**) plant biomass; (**D**) grain yield of wheat in experiment 1 (2015) and (**E**) plant height; (**F**) plant biomass; (**G**) grain yield of barley in experiment 2 (2015) in South-Eastern Australia. Error bars represent standard error; means with the same letter are not significantly different at *p* < 0.05.

When comparing the early and later BYDV-PAV-inoculated treatments to the non-inoculated control treatment, plant height was significantly reduced by both early (17%) and later (14%) infection (Figure 2A,B). Plant biomass was significantly reduced by 50% by early infection and 39% by later infection (Figure 2C). Grain yield was significantly reduced by both early and later infection; early infection reduced grain yield by 84% (1358 kg/ha) while later infection reduced grain yield by 75% (1214 kg/ha) (Figure 2D). Grain number was also significantly reduced by 84% (*p* < 0.001) by early infection and by 74% (*p* < 0.001) by later infection, while harvest index was reduced by 69% (*p* < 0.001) by early infection and by 60% (*p* < 0.001) by later infection. Thousand-grain weight was not significantly affected by either early (*p* = 0.37) or later (*p* = 0.17) infection; similarly, grain size (measured as the proportion of grain in each of the >2.8 mm, 2.5–2.8 mm, 2.2–2.5 mm, and <2.2 mm size ranges) was not significantly affected by either early (*p* ≥ 0.41) or later (*p* ≥ 0.19) infection. When comparing the early and later BYDV-PAV infection treatments to each other, there were no significant differences between early and later infection in any of the parameters measured (i.e., plant height (*p* = 0.57), plant biomass (*p* = 0.12), grain yield (*p* = 0.61), grain number (*p* = 0.54), harvest index (*p* = 0.72), 1000-grain weight (*p* = 0.87), or grain size (*p* ≥ 0.72)) (Figure S5 and Table S1). The typical virus symptom of leaf yellowing/reddening was observed in plots inoculated with BYDV-PAV, and the stunted growth of plants in the inoculated plots was obvious at harvest (Figure 2A, left). Due to the plants maturing earlier than expected as a result of the hot and dry conditions of 2015, not all of the wheat samples collected to assess BYDV-PAV incidence in each plot contained enough sap for an accurate estimation of virus incidence, however the mean incidence was at least 63% in the inoculated plots and 9% in the non-inoculated control plots, and the majority of plants in the inoculated plots were symptomatic. Additionally, a previous study showed that natural YDV background infection was low in the Wimmera region in 2015 [7]. Rainfall was 37% below average, and the mean maximum temperature was 1.1 °C above the long-term mean (Table 1).

### 3.2. Experiment 2 (2015); Barley (cv. Hindmarsh)

When comparing the early and later BYDV-PAV-inoculated treatments to the non-inoculated control treatment, plant height was not significantly affected by either early or later infection (Figure 2A,E), but plant biomass was significantly reduced by 38% by early infection and 31% by later infection (Figure 2F). Grain yield was significantly reduced by both early and later infection; early infection reduced grain yield by 60% (1352 kg/ha) while later infection reduced grain yield by 64% (1456 kg/ha) (Figure 2G). Grain number was also significantly reduced by 56% (*p* = 0.002) by early infection and 62% (*p* < 0.001) by later infection, while harvest index was reduced by 35% (*p* = 0.051) by early infection and by 47% (*p* = 0.009) by later infection. While 1000-grain weight was not significantly affected by either early (*p* = 0.06) or later (*p* = 0.18) infection, grain size was significantly affected by infection at both times; when compared to the non-inoculated control treatment, significantly more smaller grains (<2.2 mm in size) were obtained after both early (69%, *p* = 0.001) and later (59%, *p* = 0.005) infection, and significantly fewer larger grains (2.2–2.5 mm in size) were obtained after both the early (47%, *p* < 0.001) and later (38%, *p* = 0.005) infection. When comparing the early and later BYDV-PAV inoculation treatments to each other, there were no significant differences between early and later infection in any of the parameters measured (i.e., plant height (*p* = 0.82), plant biomass (*p* = 0.70), grain yield (*p* = 0.94), grain number (*p* = 0.92), harvest index (*p* = 0.66), 1000-grain weight (*p* = 0.80), or grain size (*p* ≥ 0.66)) (Figure S6 and Table S2). The typical virus symptom of leaf yellowing was observed in plots inoculated with BYDV-PAV, and the stunted growth of plants in the inoculated plots was visible at harvest (Figure 2A, right). Most of the barley samples collected to assess BYDV-PAV incidence in each plot did not contain enough sap for accurate estimation of virus incidence; however, the mean BYDV-PAV incidence was at least 20% in the inoculated plots and 3% in the non-inoculated control plots, and the majority of plants in the inoculated plots were symptomatic.

### 3.3. Experiment 3 (2017); Wheat (cv. Mace)

**Figure 3:**
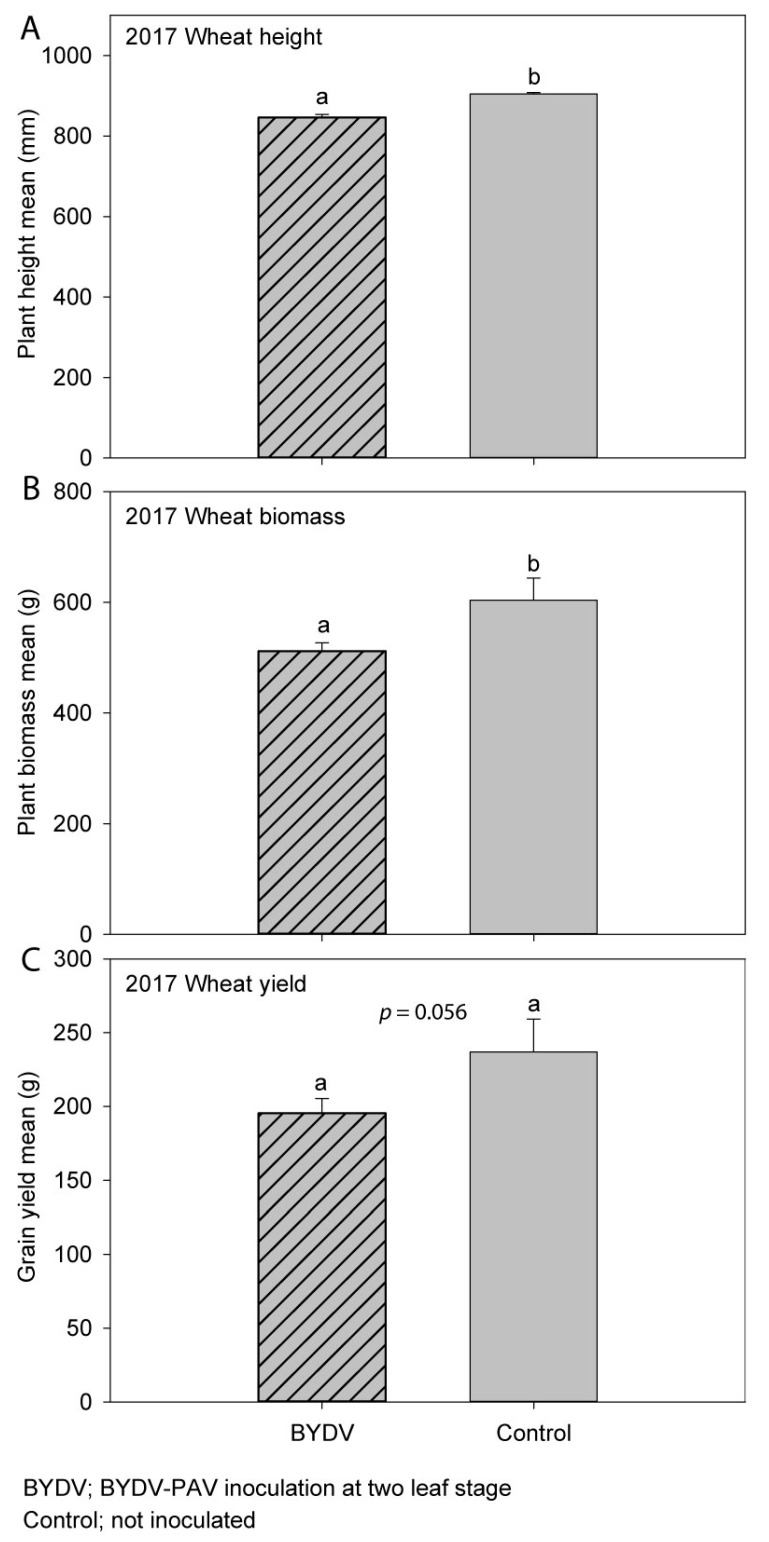
The effect of early (BYDV) or no (Control) BYDV-PAV inoculation on: (**A**) plant height; (**B**) plant biomass; (**C**) grain yield of wheat in experiment 3 (2017) in South-Eastern Australia. Error bars represent standard error; means with the same letter are not significantly different at *p* < 0.05.

**Figure 4:**
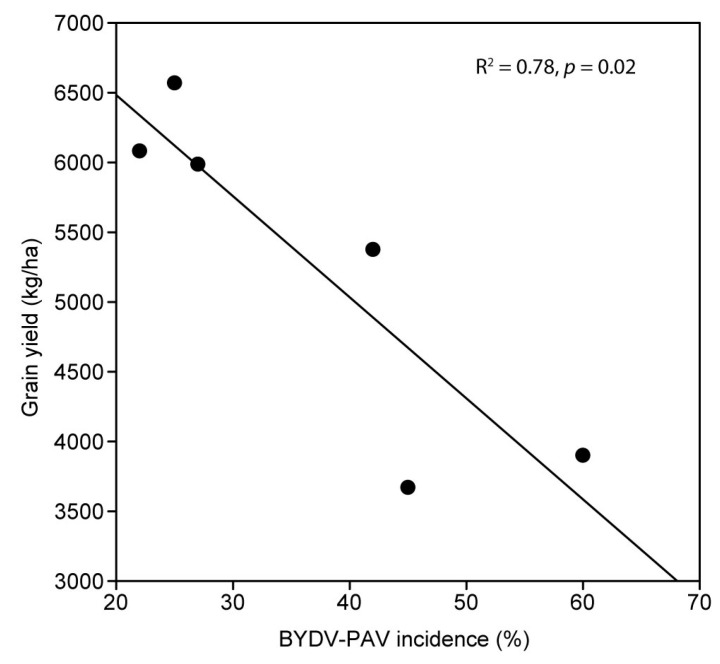
The negative linear relationship between grain yield (kg/ha) and natural background incidence of BYDV-PAV in non-inoculated control plots of wheat in experiment 3 (2017) in South-Eastern Australia revealed by regression analysis.

When comparing the BYDV-PAV-inoculated treatment to the non-inoculated control treatment, plant height was significantly reduced by 6% (Figure 3A), while plant biomass was significantly reduced by 15% (Figure 3B) due to virus infection. Grain yield was reduced by 18% (1038 kg/ha, Figure 3C), and grain number was reduced by 15% (*p* = 0.050) by virus infection, however these differences were not statistically significant at the *p* < 0.05 level. While infection did not significantly affect the number of heads per plot (*p* = 0.99), it did significantly reduce the grain weight per head (*p* = 0.04) and the number of grains per head (*p* = 0.01) by 17% and 15%, respectively. There were no significant effects of BYDV-PAV infection on harvest index (*p* = 0.35), 1000-grain weight (*p* = 0.54), grain size (*p* ≥ 0.08), grain protein (*p* = 0.43), or grain moisture (*p* = 0.20) (Figure S7 and Table S3). The typical virus symptom of leaf yellowing/reddening was observed in both inoculated and control plots (Figure 1C); however, stunted plant growth in the inoculated plots was not visually obvious at harvest. The mean BYDV-PAV incidence was 89% in the inoculated plots and 37% in the non-inoculated control plots. Linear regression analysis performed to quantify the relationship between grain yield and the unusually high level of natural BYDV-PAV background infection observed in the non-inoculated control plots revealed a negative linear relationship between the two (Pearson’s correlation coefficient R = −0.88, *p* = 0.0197) after confirming the normality of the data using the Shapiro–Wilk normality test (W statistic = 0.9185, *p* = 0.274, significance level = 0.05) (Figure 4). Grain yield decreased by 0.91% (72 kg/ha) for each 1% increase in natural BYDV-PAV background infection (Figure 4). Rainfall in Horsham was average in 2017, while the mean maximum temperature was 1.1 °C above the long-term mean (Table 1).

### 3.4. Experiment 4 (2018); Wheat (cv. Mace)

**Figure 5:**
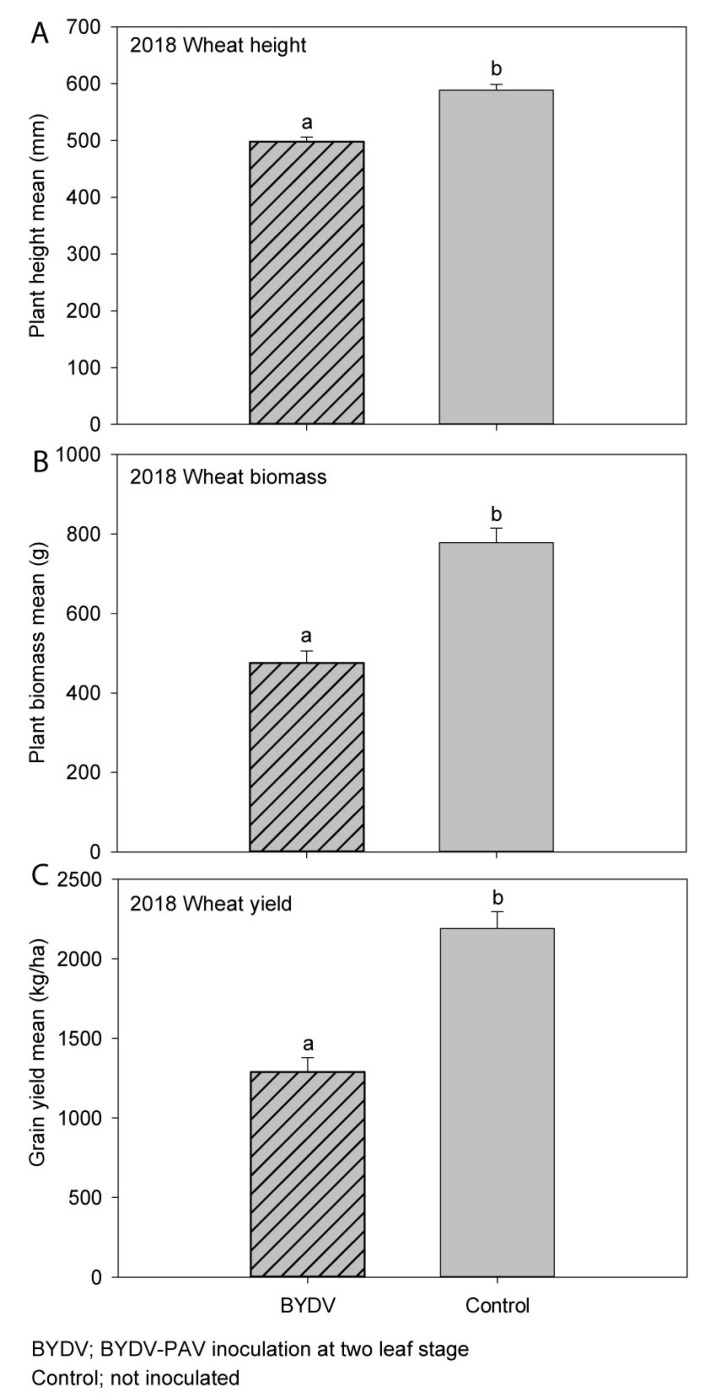
The effect of early (BYDV) or no (Control) BYDV-PAV inoculation on: (**A**) plant height; (**B**) plant biomass; (**C**) grain yield of wheat in experiment 4 (2018) in South-Eastern Australia. Error bars represent standard error; means with the same letter are not significantly different at *p* < 0.05.

When comparing the BYDV-PAV-inoculated treatment to the non-inoculated control treatment, plant height was significantly reduced by 15% (Figure 5A), while plant biomass was significantly reduced by 39% (Figure 5B) due to BYDV infection. Grain yield was reduced by 41% (923 kg/ha, Figure 5C), and grain number was reduced by 34% (*p* = 0.01) by BYD-PAV infection. While virus infection did not significantly affect the number of heads per plot (*p* = 0.15), it did significantly reduce the grain weight per head (*p* < 0.001) and the number of grains per head (*p* = 0.001) by 32% and 24%, respectively. Thousand-grain weight was significantly reduced by 10% (*p* < 0.001) by BYDV-PAV infection. Similarly, grain size was significantly affected by virus infection with 15% more smaller grains (2.5–2.8 mm in size, *p* = 0.04) and 18% fewer larger grains (>2.8 mm in size, *p* = 0.04) obtained after virus infection when compared to the non-inoculated control treatment. There were no significant effects of BYDV-PAV infection on harvest index (*p* = 0.16), grain protein (*p* = 0.07), or grain moisture (*p* = 0.81) (Figure S8 and Table S4). The typical virus symptom of leaf yellowing/reddening was observed in both inoculated and control plots, however the stunted growth of plants in the inoculated plots was not obvious at harvest. The mean BYDV-PAV incidence was 85% in the inoculated plots and 10% in the non-inoculated control plots. Rainfall in Horsham was 44% below average in 2018 while the mean maximum temperature was 1.5 °C above the long-term mean (Table 1).

## 4. Discussion

This study quantified yield losses associated with BYDV-PAV infection in cereals grown under field conditions in South-Eastern Australia. In four experiments conducted over three years, we report yield reductions of up to 84% (1358 kg/ha) in wheat and 64% (1456 kg/ha) in barley, along with an estimated yield loss impact factor of 0.91% (72 kg/ha) for each one percent increase in natural BYDV-PAV infection in wheat. Although a previous study of yield losses associated with an isolate of CYDV-RPV in Victoria was published more than 30 years ago [24] and showed yield losses of up to 79% as a result of virus infection, our study quantifies yield losses associated with BYDV-PAV, the most prevalent YDV species found in cereal fields in the region [7]. Despite the implementation of the latest control strategies, grain yield and quality losses resulting from virus infection are still common and often devastating [30]; however, the impact of viruses on cereal production in South-Eastern Australia is still often underestimated. In some years, YDVs are prevalent and can occur with high incidences in cereals in South-Eastern Australia [7]. Although the losses reported in this study were the result of artificially introduced aphids, they demonstrate the severe yield losses that can result from BYDV-PAV infection in the region, particularly when high numbers of viruliferous aphids are present. Combined with the recently updated YDV incidence data [7], this yield loss information will assist with the provision of a more accurate understanding of the impacts of YDV infection in currently grown cereal cultivars in South-Eastern Australia.

Yield losses due to BYDV-PAV infection were recorded each year of the experiment but were particularly severe and obvious in the hot and dry year of 2015, where virus infection severely reduced already low yields. While severe yield losses such as these have previously been reported in cereals [13,16,18,20,31,32], the same severe losses were not observed in our experiments in 2017 or 2018; however, the 41% yield loss recorded in 2018 (experiment 4) was still severe and similar to those reported by others [14,22,23,24,25]. The high level of natural BYDV-PAV background infection present in the non-inoculated control plots in wheat in 2017 (experiment 3) is likely to have reduced the difference between the inoculated and non-inoculated treatments, thereby masking the severity of yield losses resulting from BYDV-PAV infection.

Linear regression analysis of grain yield with natural BYDV-PAV background infection detected in the non-inoculated control plots revealed a negative linear relationship between the two, showing a decrease in yield of 0.91% (72 kg/ha) for each 1% increase in natural BYDV-PAV infection; similar negative relationships between yield loss and BYDV incidence were reported by others [16,17,21,23,24]. The lowest and highest incidence levels used for regression analysis were 22% and 60%, respectively, so any predictions based on incidence levels outside of this range would be based on extrapolation; also, it is not known if linearity still applies to the relationship when incidences are outside of this range. It is not known when the background infection occurred, and other YDV species may also have been present in the non-inoculated control plots so the incidences used for regression analysis may be a slight underestimation. 

The effects of early (inoculated at the seedling stage) and later (inoculated at early tillering) inoculation with BYDV-PAV was assessed in wheat (experiment 1) and barley (experiment 2) in 2015. Significant reductions in yield, grain number, plant biomass, and harvest index were recorded after both early and later infection, in both wheat and barley, when compared to the non-inoculated control plots. Additionally, there was no statistically significant difference between early and later BYDV-PAV infection in grain yield or any other parameter measured (grain number, 1000-grain weight, grain size, plant height, biomass, and harvest index) in either wheat or barley when the two inoculation times were compared to each other, showing that significant yield losses can occur in wheat and barley following both early and later infection. While the majority of BYDV yield loss studies report greater yield losses from early BYDV infection and that yield losses associated with late infection are not significant [23,24], other studies have reported lower but significant yield losses from late inoculation, particularly in susceptible cultivars [11,14,16], while others have reported no significant differences in yield between early and later inoculation [15,16,22]. This variation seems to be influenced by factors such as cultivar, virus isolate, time of inoculation, and environmental conditions, among others. In this study, the early inoculation was done at the seedling stage (Z12–13) and the later inoculation was done at early-tillering (Z21–22), while in a number of other studies, late inoculation was done at or after early stem extension (Z30) [22,24]. Therefore, our later inoculation was applied at a relatively early growth stage in comparison to some other studies. 

In each experiment, yield losses resulting from BYDV-PAV infection were accompanied by similar-sized reductions in grain number per plot while 1000-grain weight and grain size were affected to a lesser extent and differences were only significant in some experiments, indicating that the yield losses were primarily due to the presence of less grain rather than smaller grain. Furthermore, virus infection did not significantly affect the number of heads per plot but did significantly reduce the number of grains per head and the weight of grain from each head. Thousand-grain weight and grain size were not significantly affected by infection in wheat in experiment 1 (despite the severe yield loss observed) or experiment 3, but were significantly affected in experiment 4. In cereals, the number of tillers per plant [14,24,33], heads per meter, seeds per head, along with 1000-grain weight [15,16,31,34], number of heads with aborted terminal spikelets [24], and grain size/quality [17,25] have all been reported to be significantly affected by BYDV infection in some hosts and/or cultivars. Although leaf symptoms typical of BYDV infection were observed in each experiment, stunted plant growth was only obvious at harvest in experiments 1 and 2 (Figure 2A,E) and was not noticeable at harvest in experiments 3 or 4, despite the 39% reduction in biomass and 41% yield loss recorded in experiment 4. The reduced visibility of the effects of BYDV infection at harvest in experiment 4 suggests that infection at these levels is unlikely to be noticed in the field, which in turn is likely to have contributed to the continued underestimation of the importance of BYDV noted in previous studies [21]. It is not clear why the effects of BYDV infection were so much more obvious in wheat in experiment 1 than experiment 4. Plant height and biomass were reduced by a similar amount in each experiment, however harvest index (the ratio of grain yield to above-ground plant biomass) calculations show that BYDV infection affected yield more than plant biomass in experiment 1 but not in experiments 3 or 4. Differences in factors such as growing conditions and cultivar, among others, are likely to have contributed to the difference in harvest index between 2015 and 2018. 

The effects of BYDV infection on parameters such as yield, grain number, grain size, grain weight, harvest index, and symptom expression vary between experiments and studies, and can be influenced by several factors. Environmental factors such as soil moisture, rainfall, and temperature, and their effects on plants, vectors, and viruses, are likely to have contributed to the variation in results between studies and experiments. While it has been suggested that plants infected with BYDV and other viruses may be more drought tolerant than non-infected plants [35,36,37], the results of this study do not support these findings. While describing BYDV in 1953, Oswald and Houston [11] noted that damage caused by BYDV infection was particularly severe in a drought year, and others have also reported more severe yield losses from BYDV infection in years of lower rainfall [15,25,38,39]. Given that the root system of a plant infected with BYDV is also often just as stunted, and sometimes even more so, than the visible, above-ground portion of the plant [11,40,41,42,43], it has been suggested that the roots of infected plants may be too shallow to reach or obtain adequate water and nutrients in dry conditions [11]. This is one possible explanation for the especially severe yield losses observed in experiment 1, as 2015 was the second year in a row of well below average rainfall in the region (Table 1).

Differences in factors such as YDV species/isolate, host cultivar, time of infection, and aphid vector species are also likely to have contributed to the variation in results between studies and experiments. For example, when Monneveux et al. [39] reported higher yield losses due to BYDV infection in the drought year of 1988 than the average rainfall year of 1987, they also reported that the same severe yield losses were not observed in lines that were tolerant to BYDV. Others have also reported significant differences in the response of different cultivars to BYDV infection, especially between susceptible and resistant cultivars [13,14,25]. Given that different wheat cultivars were used in 2015 and 2018, this is also likely to have contributed to the different results obtained in those years. Furthermore, Baltengberger et al. [13] reported more severe symptoms and greater yield losses in plants infected with both BYDV-PAV and CYDV-RPV than they did in plants inoculated with one isolate or the other singly. A high level of *Septoria tritici* was also observed in experiment 3 and may also have played a role, as significant interactions between BYDV and other diseases have been reported [44,45]. Thus, some of the yield loss reported here may be attributed to other factors that have not been analyzed or captured here, but some of which have been discussed.

In conclusion, the four randomized, replicated field experiments conducted in this study quantified the yield losses associated with BYDV-PAV infection in cereals grown in South-Eastern Australia. The results of these experiments demonstrate the potential for severe yield losses that can result from infection with BYDV-PAV, the most prevalent species of BYDV in South-Eastern Australia, showing yield losses of up to 84% (1358 kg/ha) in wheat and 64% (1456 kg/ha) in barley due to BYDV infection. Additionally, an estimated yield loss impact factor of 0.91% (72 kg/ha) for each one percent increase in natural BYDV-PAV infection was obtained for wheat. Yield losses varied between experiments and years, demonstrating that losses can be influenced by many factors, such as cereal cultivar and environmental factors, and illustrating the importance of conducting these experiments under varying conditions. The results of this study will assist with the provision of more accurate estimates of current yield losses in cereals due to BYDV infection and a more accurate understanding of the importance of BYDV in Victoria and more broadly in South-Eastern Australia.

## Figures and Tables

**Table 1:** Three-monthly annual rainfall (mm) and mean maximum temperature (°C) for the years 2014–2018 and three-monthly long-term mean rainfall (mm) and long-term mean maximum temperature (°C) for the years 1961–2018 for the Wimmera region, South-Eastern Australia.

	Year	Jan–Mar	Apr–Jun	Jul–Sep	Oct–Dec
Rainfall (mm)	2014	36	133	64	33
	2015	88	77	60	28
	2016	69	110	222	108
	2017	56	135	128	112
	2018	24	82	79	42
Long-term mean	1961–2018	67	107	132	97
Mean maximum temperature (°C)	2014	30.9	18.4	16.4	27.3
	2015	29.1	17.1	15.4	29.3
	2016	30.2	18.4	14.8	24.0
	2017	30.3	17.9	15.3	27.2
	2018	30.6	19.0	16.0	26.7
Long-term mean	1961–2018	28.9	17.8	15.2	24.5

## Supplementary Materials

The following are available online at https://www.mdpi.com/2076-2607/9/3/645/s1, Figure S1: Quantile–quantile plots to assess normality in experiment 1 (wheat, 2015); Figure S2: Quantile–quantile plots to assess normality in experiment 2 (barley, 2015); Figure S3: Quantile–quantile plots to assess normality in experiment 3 (wheat, 2017); Figure S4: Quantile–quantile plots to assess normality in experiment 4 (wheat, 2018); Figure S5: Percent change associated with early and later BYDV infection in experiment 1 (wheat, 2015); Table S1: *p*-Values calculated according to Student’s *t*-tests or ANOVA for experiment 1 (wheat, 2015); Figure S6: Percent change associated with early and later BYDV infection in experiment 2 (barley, 2015); Table S2: *p*-Values calculated according to Student’s *t*-tests or ANOVA for experiment 2 (barley, 2015); Figure S7: Percent change associated with early BYDV infection in experiment 3 (wheat, 2017); Table S3: *p*-Values calculated according to Student’s *t*-tests or ANOVA for experiment 3 (wheat, 2017); Figure S8: Percent change associated with early BYDV infection in experiment 4 (wheat, 2018); Table S4: *p*-Values calculated according to Student’s *t*-tests or ANOVA for experiment 4 (wheat, 2018).
