# Supplementary material for: Yield Losses Caused by Barley Yellow Dwarf Virus-PAV Infection in Wheat and Barley: A Three-Year Field Study in South-Eastern Australia
Source: Microorganisms. 2021 Mar 19;9(3):645. doi: 10.3390/microorganisms9030645 (PMC8003756; doi:10.3390/microorganisms9030645)
Supplement: Supplementary file 1 [file microorganisms-09-00645-s001.zip › microorganisms-1128562 suppl final.pdf]

microorganisms

MDPI

Supplementary Materials

Yield Losses Caused by Barley Yellow Dwarf Virus-PAV Infection in Wheat and Barley: A Three-Year Field Study in South-Eastern Australia

Narelle Nancarrow [1,*], Mohammad Aftab [1], Grant Hollaway [1], Brendan Rodoni [2,3] and Piotr Trebicki [1,4]

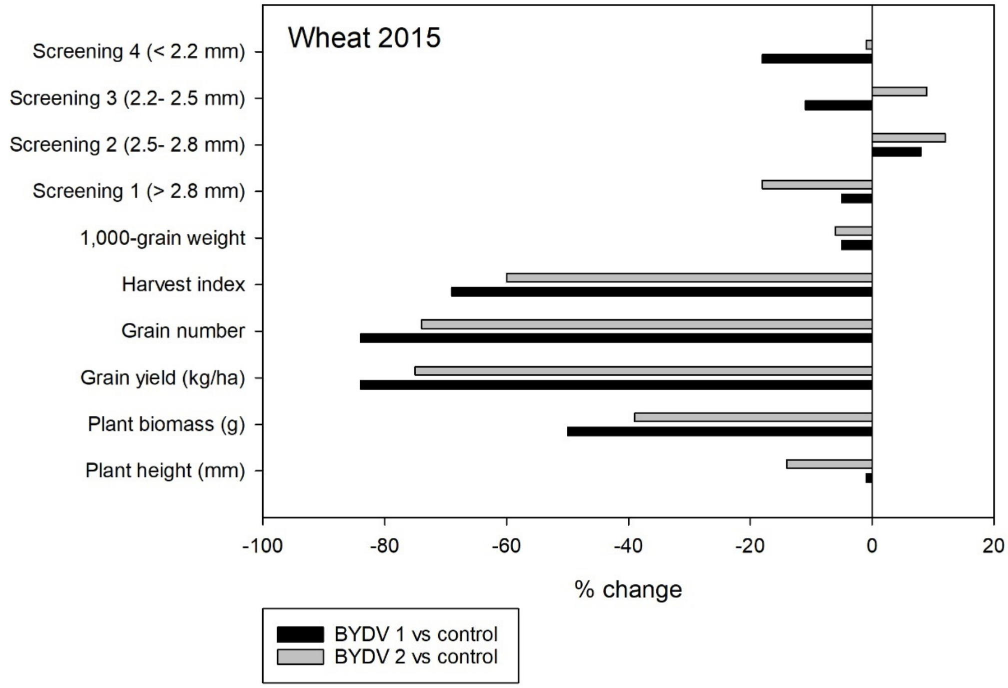

Figure S5. Percent change associated with early (BYDV 1) and later (BYDV 2) inoculation treatments in comparison to the control treatment for grain and plant parameters measured in wheat in experiment 1 (2015). See Table S1 for the *p*-values calculated according to Student's *t*-tests or ANOVA.

Table 1. *p*-Values calculated according to Student's *t*-tests or ANOVA for each comparison between the three treatments (BYDV-1, BYDV-2, and control) for each parameter measured in wheat in experiment 1 (2015). Screenings represent grain size, measured as the proportion of grain in each of the > 2.8 mm, 2.5–2.8 mm, 2.2–2.5 mm, and < 2.2 mm size ranges.

**Wheat 2015**	**BYDV 1 vs. Control**	**BYDV 2 vs. Control**	**BYDV 1 vs. BYDV 2**
Screening 4 (<2.2 mm)	$p = 0.88$	$p = 0.9996$	$p = 0.90$
Screening 3 (2.2–2.5 mm)	$p = 0.94$	$p = 0.95$	$p = 0.80$
Screening 2 (2.5–2.8 mm)	$p = 0.41$	$p = 0.19$	$p = 0.86$
Screening 1 (>2.8 mm)	$p = 0.96$	$p = 0.55$	$p = 0.72$
1000-grain weight	$p = 0.37$	$p = 0.17$	$p = 0.87$
Harvest index	$p < 0.001$	$p < 0.001$	$p = 0.72$
Grain number	$p < 0.001$	$p < 0.001$	$p = 0.54$
Grain yield (kg/ha)	$p < 0.001$	$p < 0.001$	$p = 0.61$
Plant biomass (g)	$p < 0.001$	$p < 0.001$	$p = 0.12$
Plant height (mm)	$p < 0.001$	$p < 0.001$	$p = 0.57$

Copyright: © 2021 by the authors. Licensee MDPI, Basel, Switzerland. This article is an open access article distributed under the terms and conditions of the Creative Commons Attribution (CC BY) license (http://creativecommons.org/licenses/by/4.0/).

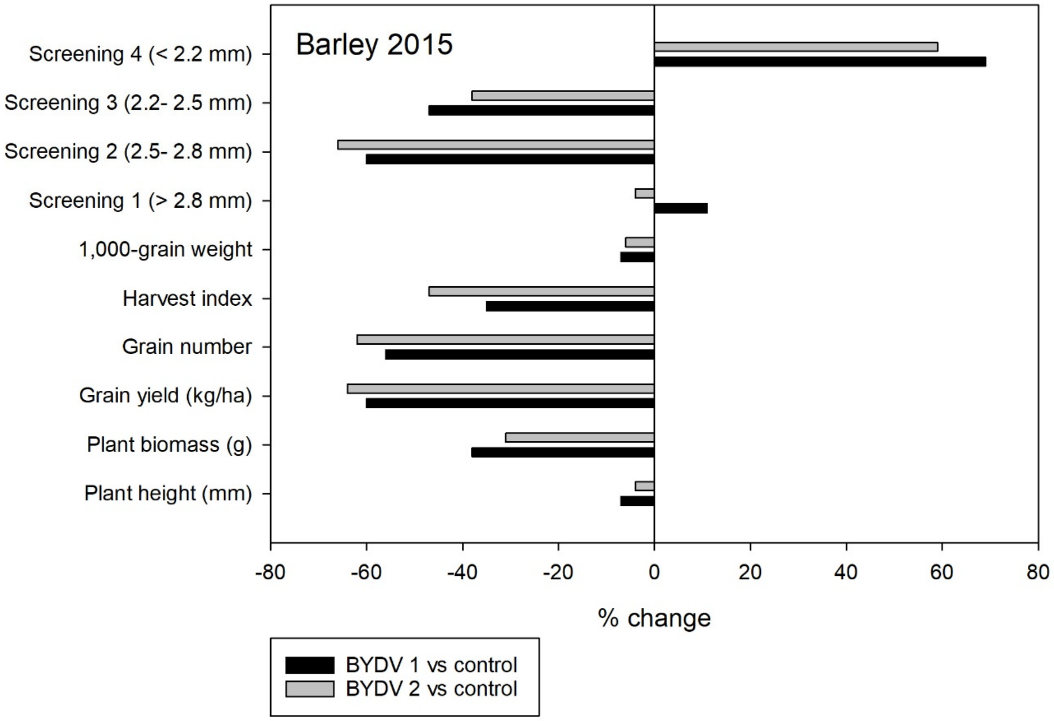

Figure S6. Percent change associated with early (BYDV 1) and later (BYDV 2) inoculation treatments in comparison to the control treatment for grain and plant parameters measured in barley in experiment 2 (2015). See Table S2 for the *p*-values calculated according to Student's *t*-tests or ANOVA.

Table 2. *p*-Values calculated according to Student's *t*-tests or ANOVA for each comparison between the three treatments (BYDV-1, BYDV-2, and control) for each parameter measured in barley in experiment 2 (2015). Screenings represent grain size which were measured as the proportion of grain in each of the > 2.8 mm, 2.5–2.8 mm, 2.2–2.5 mm, and < 2.2 mm size ranges.

Barley 2015	BYDV 1 vs. Control	BYDV 2 vs. Control	BYDV 1 vs. BYDV 2
Screening 4 (<2.2 mm)	$p = 0.001$	$p = 0.005$	$p = 0.79$
Screening 3 (2.2–2.5 mm)	$p < 0.001$	$p = 0.005$	$p = 0.66$
Screening 2 (2.5–2.8 mm)	$p = 0.056$	$p = 0.034$	$p = 0.97$
Screening 1 (>2.8 mm)	$p = 0.82$	$p = 0.97$	$p = 0.68$
1000-grain weight	$p = 0.057$	$p = 0.17$	$p = 0.17$
Harvest index	$p = 0.051$	$p < 0.001$	$p = 0.66$
Grain number	$p = 0.002$	$p < 0.001$	$p = 0.92$
Grain yield (kg/ha)	$p = 0.0014$	$p < 0.001$	$p = 0.94$
Plant biomass (g)	$p = 0.007$	$p < 0.001$	$p = 0.70$
Plant height (mm)	$p = 0.40$	$p = 0.76$	$p = 0.81$

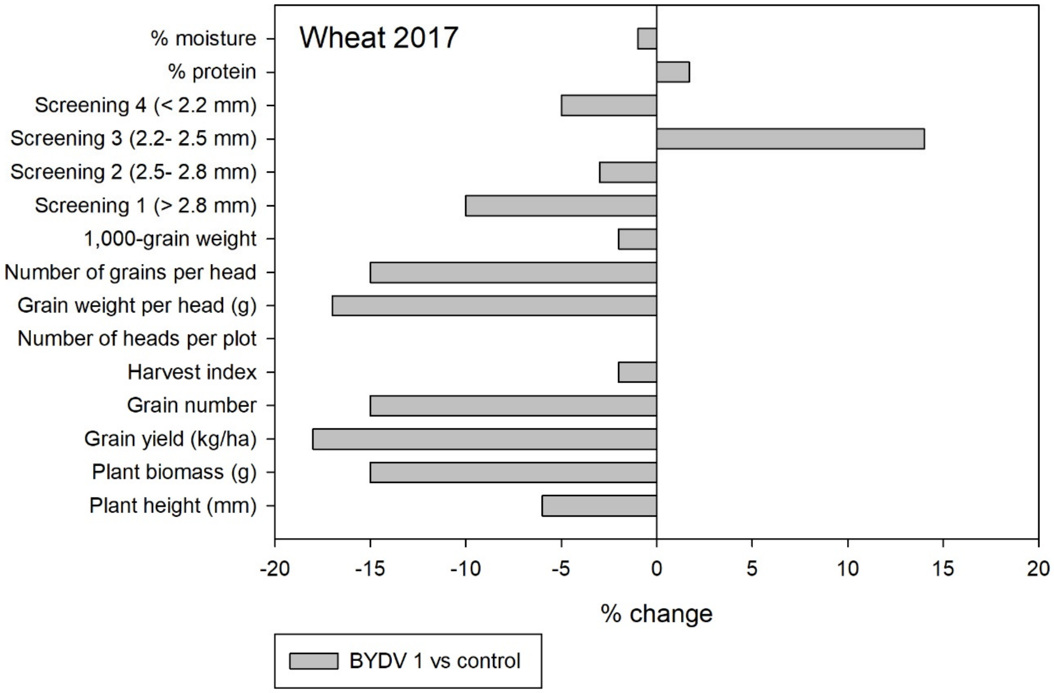

Figure S7. Percent change associated with early (BYDV 1) and later (BYDV 2) inoculation treatments in comparison to the control treatment for grain and plant parameters measured in wheat in experiment 3 (2017). See Table S3 for the *p*-values calculated according to Student's *t*-tests or ANOVA.

Table 3. *p*-Values calculated according to Student's *t*-tests or ANOVA for each comparison between the two treatments (BYDV 1 and control) for each parameter measured in wheat in experiment 3 (2017). Screenings represent grain size which were measured as the proportion of grain in each of the >2.8 mm, 2.5–2.8 mm, 2.2–2.5 mm, and <2.2 mm size ranges.

Wheat 2017	BYDV vs. Control
% moisture	$p = 0.20$
% protein	$p = 0.43$
Screening 4 (<2.2 mm)	$p = 0.80$
Screening 3 (2.2–2.5 mm)	$p = 0.08$
Screening 2 (2.5–2.8 mm)	$p = 0.62$
Screening 1 (>2.8 mm)	$p = 0.35$
1000-grain weight	$p = 0.54$
Number of grains per head (g)	$p = 0.009$
Grain weight per head (g)	$p = 0.035$
Number of heads per plot	$p = 0.99$
Harvest index	$p = 0.35$
Grain number	$p = 0.0499$
Grain yield (kg/ha)	$p = 0.056$
Plant biomass (g)	$p = 0.048$
Plant height (mm)	$p = 0.001$

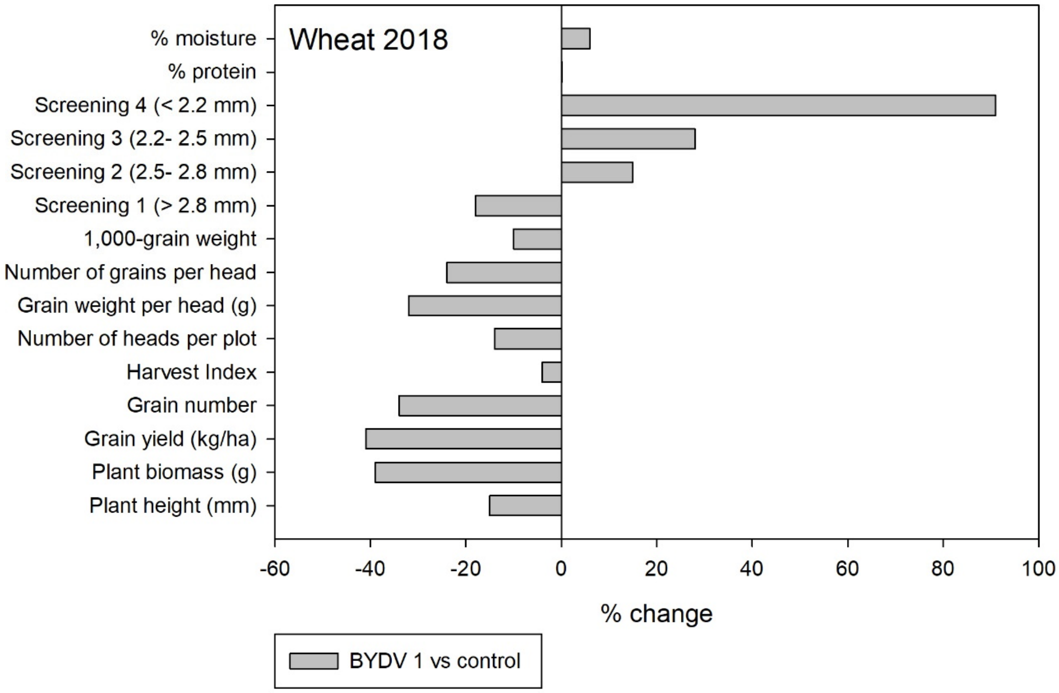

Figure S8. Percent change associated with early (BYDV 1) and later (BYDV 2) inoculation treatments in comparison to the control treatment for grain and plant parameters measured in wheat in experiment 4 (2018). See Table S4 for the *p*-values calculated according to Student's *t*-tests or ANOVA.

Table 4. *p*-Values calculated according to Student's *t*-tests or ANOVA for each comparison between the two treatments (BYDV 1 and control) for each parameter measured in wheat in experiment 4 (2018). Screenings represent grain size which were measured as the proportion of grain in each of the >2.8 mm, 2.5–2.8 mm, 2.2–2.5 mm, and <2.2 mm size ranges.

Wheat 2018	BYDV vs. Control
% moisture	$p = 0.81$
% protein	$p = 0.070$
Screening 4 (<2.2 mm)	$p = 0.045$
Screening 3 (2.2–2.5 mm)	$p = 0.17$
Screening 2 (2.5–2.8 mm)	$p = 0.038$
Screening 1 (>2.8 mm)	$p = 0.036$
1000-grain weight	$p < 0.001$
Number of grains per head (g)	$p = 0.0043$
Grain weight per head (g)	$p < 0.001$
Number of heads per plot	$p = 0.15$
Harvest index	$p = 0.16$
Grain number	$p = 0.013$
Grain yield (kg/ha)	$p = 0.005$
Plant biomass (g)	$p = 0.004$
Plant height (mm)	$p < 0.001$
